# Return to work within 2 years of lumbar fusion: a prospective cohort study

**DOI:** 10.2340/17453674.2025.43751

**Published:** 2025-08-15

**Authors:** Jenna L C LAURÉN, Leevi A TOIVONEN, Jussi P REPO, Hannu KAUTIAINEN, Arja H HÄKKINEN, Marko H NEVA

**Affiliations:** 1 Department of Orthopaedics and Trauma, Tampere University Hospital, Tampere; 2 Faculty of Medicine and Health Technology, Tampere University, Tampere; 3 Primary Health Care Unit, Kuopio University Hospital, Kuopio; Folkhälsan Research Center, Helsinki; 4 Biology of Physical Activity, Faculty of Sport and Health Sciences, University of Jyväskylä, Jyväskylä; 5 Health Sciences, Faculty of Sport and Health Sciences, University of Jyväskylä, Jyväskylä, Finland

## Abstract

**Background and purpose:**

Return to work is an important objective and measure of treatment success in the working-age population. Many patient-related factors have been shown to be associated with failure to resume working postoperatively. The aim of this longitudinal database study was to determine return to work rates in a 24-month follow-up after lumbar fusion. We also evaluated whether return to work was affected by the physical demand of work or the preoperative dominance of leg or back pain.

**Methods:**

348 consecutive patients available to the workforce underwent lumbar fusion. Return to work at 12 and 24 months was evaluated by patient questionnaires. Patients rated the physical demand of work into 3 categories: light, moderately demanding, or demanding. The surgeon identified the predominant symptom preoperatively, dividing patients into back and leg pain groups.

**Results:**

Return to work was 69% (95% confidence interval [CI] 64–73) and 76% (CI 71–81), at 12- and 24-month follow-ups, respectively. Patients in physically demanding work were less likely to resume working than patients in light work (63% vs 86% at 24 months, respectively). The predominant symptom did not affect return to work.

**Conclusion:**

In patients of working age, three-quarters of lumbar spine fusion patients returned to work within 2 years of surgery. Work absenteeism was higher in physically demanding occupations and only 60% of the patients with predominant leg pain returned to their physically heavy occupation in the first year following lumbar fusion.

Return to work is both an aim of surgery and an important measure of its success. After lumbar spine fusion, return to work varies from 43% to 90% depending on patient selection, patient comorbidities and preoperative work status [1-3]. Work absenteeism causes productivity loss, which inflicts a high financial burden on the individual patient [4-6] and the economy [6,7]. Work presenteeism (reduced at-work performance) also causes indirect losses, but is harder to quantify [8]. Reduced work absenteeism and presenteeism have been reported after single-level lumbar spine surgery [9]. Working at the time of surgery has been linked to favorable results after spinal fusion [10,11]. In fact, disengagement in work preoperatively is a strong risk factor for not resuming work after lumbar spine surgery [1,3,12,13].

Physically demanding work is also associated with reduced rates of return to work [12,14,15] and other inferior outcomes [16] following spinal surgery. Severe preoperative back pain has been associated with a lower likelihood of return to work after elective lumbar spine surgery [12,14,17,18]. After surgery for degenerative spondylolisthesis and spinal stenosis, patients with predominant leg pain improved more than those with predominant back pain [19]. With increasing lumbar fusion rates [20-22], securing sufficient return to work rates is essential, especially in younger patients.

The aim of this study is to evaluate return to work in a 2-year follow-up after lumbar spine fusion, and to investigate the influence of self-rated physical demands of work and predominance of back or leg pain on return to work.

## Methods

### Study design, setting and participants

The present prospective cohort study included patients available to the workforce (18–65 years), and patients on a disability pension at the time of surgery were excluded from the study. Other exclusion criteria were surgery for tumor or acute fracture. Patients with a history of previous fusion surgery were included in the study. The STROBE reporting guideline was followed [23].

During the years 2008–2014 a database was established, including consecutive patients undergoing elective lumbar spine fusion in Tampere University Hospital and Central Finland Central Hospital. During years 2008–2014, information on 817 patients was available. At the time of surgery these 2 tertiary hospitals were the only units performing lumbar spine fusion in their geographical catchment area of 775,000 inhabitants. Patients were recruited at the outpatient clinic by the orthopedic surgeon after the decision for surgery. The indication for surgery, predominant symptoms, and surgical details were filled in by the surgeon at the time of surgery. Clinical postoperative visits were arranged according to normal clinical practice, with postoperative visits to the surgeon 3 months and 1 year after surgery and later, if necessary, until the postoperative situation stabilized. Cumulative return to work was retrospectively collected from patient records with no missing data.

“All surgeries” included pedicle screw instrumentation and decompression via a posterior, midline approach. Interbody devices were used at the surgeon’s discretion. During the study period, a common practice was to issue a medical certificate allowing sick leave for 3 months postoperatively, and the sick leave was continued as needed.

### Variables

Patient-reported outcome measures (PROMs) were collected via questionnaires at baseline, and at 3, 12, and 24 months postoperatively. These included the visual analogue scale (VAS) for back and leg pain, ranging from 0 to 100 mm, higher scores indicating greater pain [24]. The Oswestry Disability Index (ODI) [25,26], ranging from 0 to 100 with higher scores indicating more severe disability, was also included.

Job titles from the baseline questionnaires were transformed into socioeconomic statuses based on the Classification of Occupations 1989 in Finland [27] (Table 1). The classification defines the groups as manual workers, self-employed persons, and upper- or lower-level employees. The main difference with upper- and lower-level employees is that lower-level employees operate at the execution level of an organization and require less theoretical knowledge, whereas upper-level employees have a higher position in the decision-making hierarchy and the need for more extensive theoretical knowledge. Patients also rated their work according to physical demand into 3 categories: light, moderately demanding, and demanding work. Light work was described as sedentary or light standing work, for example desk jobs. Moderately demanding work was described as heavy sedentary work or moderately demanding work, requiring more physical activity, such as teaching or cleaning jobs. Demanding work included both heavy and extremely heavy physical work, for example construction jobs. Patients were stratified into back pain and leg pain groups according to surgeon-determined preoperative dominant complaint.

**Table 1:** Patient demographics and clinical data. Values are count (%) unless otherwise specified

Factor	Leg pain n = 246	Back pain n = 102
Women	138 (56)	63 (62)
Age, years, mean (SD)	51 (8)	47 (9)
Education, years, mean (SD)	13.2 (3.4)	13.5 (3.1)
Smoking	58 (24)	18 (18)
Units of alcohol/week, median (IQR)	2.0 (0.0–8.0)	1.5 (0.0–5.0)
Cohabiting ^**a**^	185 (75)	73 (72)
Body mass index, mean (SD)	27.3 (4.2)	27.1 (3.8)
Socioeconomic group		
Self-employed persons	18 (7.3)	6 (5.9)
Upper-level employees	38 (15)	14 (14)
Lower-level employees	106 (43)	47 (46)
Manual workers	84 (34)	35 (34)
Self-rated physical demand of work		
Light	102 (41)	47 (46)
Medium	69 (28)	26 (25)
Heavy	75 (30)	29 (28)
Sick leave, days, median (IQR) ^**b**^	90 (5–240)	90 (14–210)
Duration of the spinal problem, years, median (IQR)	8.5 (3–15)	10.5 (4–20)
VAS back pain, mean (SD)	62 (25)	68 (21)
VAS leg pain, mean (SD)	67 (22)	50 (28)
Oswestry Disability Index, mean (SD)	44 (15)	42 (13)
Comorbidities		
Cardiovascular	97 (39)	28 (27)
Diabetes	22 (8.9)	2 (2.0)
Rheumatoid	5 (2.0)	2 (2.0)
Neurological	4 (1.6)	3 (2.9)
Psychiatric	8 (3.3)	2 (2.9)
Respiratory	6 (2.4)	4 (3.9)
Indication for surgery		
Degenerative spondylolisthesis	101 (41)	18 (18)
Isthmic spondylolisthesis	69 (28)	34 (33)
Degenerative disc disease (DDD)	26 (11)	25 (25)
Spinal stenosis (LSS)	29 (12)	7 (6.9)
Deformity	8 (3.3)	10 (10)
Postoperative conditions	13 (5.3)	8 (7.8)
Previously undergone spinal surgery	70 (30)	25 (25)
Previously undergone spinal fusion	20 (23)	3 (2.9)
Fusion length, levels		
1–2	217 (88)	85 (83)
> 2	29 (12)	17 (17)

a Domestic relationship or marriage.

b Sick leave days during the last 12 months prior to surgery.

SD = standard deviation; IQR = interquartile range, VAS = visual analogue scale.

### Statistics

Summary statistics were described using mean and standard deviation (SD), median and interquartile range (IQR), or numbers and percentages. Factors associated with return to work at 24-month follow-up were estimated using a multiple Cox regression model. Proportional hazards assumption was also checked by examining plots of the scaled Schoenfeld residuals against time for the covariates. We used the Kaplan–Meier method to illustrate cumulative risk of return to work for the groups. The log-rank test was applied to test the differences and trend of the cumulative function of return to work across self-rated physical demands of work (ordered groups). P-value < 0.05 was considered statistically significant. Stata 18 (StataCorp LLP, College Station, TX, USA) was used for the analysis.

### Ethics, funding, use of AI tools, and disclosures

All participants gave written informed consent for participation in the study. The study has received approval from the Ethical Boards of Tampere University Hospital and Central Finland Central Hospital (approval number R07108). This study was financially partly supported by the State funding for university-level health research, Tampere University Hospital, Wellbeing Services, county of Pirkanmaa. ChatGPT was used in text editing for the introduction and discussion sections. The authors report no conflicts of interest. Complete disclosure of interest forms according to ICMJE are available on the article page, doi: 10.2340/17453674.2025.43751

## Results

**Figure 1:**
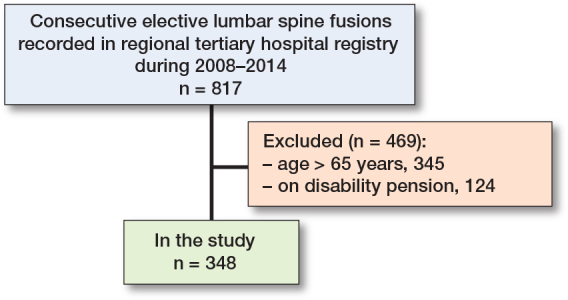
Patient flowchart.

13 patients did not consent and were not included in the database (n = 817) (Table 1 and Figure 1). 469 patients were excluded, 345 because of age (over 65 years) and 124 because they were on a pension. 348 patients were available to the workforce (age 18–65) and were included in the study. Of these patients, 190 (55%) were working preoperatively. The mean age of the patients at the time of surgery was 50 years, and 58% were women. The median duration of the spinal symptoms was 10 years. Almost half of the patients (44%) were low-level employees and one-third of the patients (30%) considered their work to be physically heavy. Main indications for surgery were degenerative spondylolisthesis (34%) or isthmic spondylolisthesis (30%). All patients underwent posterolateral instrumented fusion, where most of the fusions (87%) were short (1–2 levels). 246 patients (71%) were allocated to the leg pain group, and 102 patients (29%) to the back pain group. Patients in the leg pain group were older and had more comorbidities. Patients in the back pain group suffered from symptoms for a longer time (see Table 1).

### Return to work

The overall return to work rate at 12 months was 69% (95% confidence interval [CI] 64–73) and at 24 months it was 76% (CI 71–81). At 12-month follow-up 68% (CI 62–73) of the leg pain group and 73% (CI 65–82) of the back pain group had returned to work. At 24-month follow-up, return to work was 77% (CI 71–82) for the leg pain group and 76% (CI 67–84) for the back pain group, showing similar rates of return to work between the groups (HR [adjusted for age, sex, diagnosis, and self-rated physical demands of work] = 1.02, CI 0.77–1.36). At 24-month follow-up, 94% (CI 90–97) of patients who were working preoperatively, and 55% (CI 48–63) of patients who were not working preoperatively, returned to work (P < 0.001).

**Figure 2:**
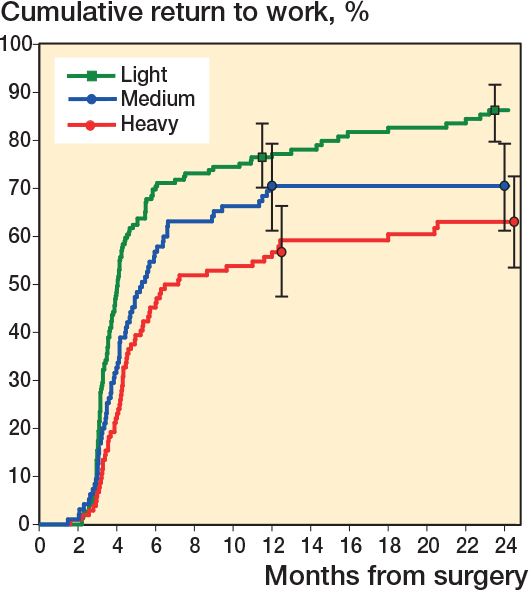
Cumulative return to work for all patients (both leg pain and back pain dominant symptoms) according to self-rated physical demands of work. P value for trend (< 0.001) was adjusted for age, sex, and diagnosis.

Patients in self-rated physically demanding work were less likely to resume working than patients in physically medium or light work (Figure 2). Return to work at 24 months was 86% (CI 80–92) for patients in light work, 75% (CI 66–84) for patients in medium work, and 63% (CI 54–73) for patients in heavy work (adjusted P for trend < 0.001).

### Factors associated with return to work

In the leg pain group, the likelihood of return to work was higher in upper-level employees, and in patients with lower ODI scores. Patients in the leg pain group who had undergone previous surgery and patients in manual work were less likely to return to work. In the back pain group, the likelihood of return to work was higher among patients under 50 years old and in upper-level employees (Table 2).

**Table 2:** Factors associated with return to work at 24-month follow-up by a multiple Cox regression model

Factor	Leg pain n = 246 HR (CI)	Back pain n = 102 HR (CI)
Male sex	0.83 (0.38–1.28)	0.74 (0.36–1.51)
Age ≥ 50	0.73 (0.50–1.06)	0.54 (0.31–0.93) ^**a**^
Socioeconomic group		
Lower-level employees	Reference	Reference
Self-employed persons	0.85 (0.45–1.60)	2.18 (0.74–6.42)
Upper-level employees	1.77 (1.10–2.83) ^**a**^	2.72 (1.19–6.24) ^**a**^
Manual workers	0.56 (0.37–0.86) ^**a**^	0.86 (0.42–1.76)
Duration of the spinal problem	1.00 (0.99–1.02)	1.01 (0.97–1.01)
Oswestry Disability Index	0.98 (0.97–0.99) ^**a**^	0.99 (0.97–1.01)
Indication for surgery		
Degenerative spondylolisthesis	Reference	Reference
Isthmic spondylolisthesis	0.99 (0.66–1.50)	0.76 (0.35–1.65)
Degenerative disc disease	1.20 (0.68–2.11)	0.67 (0.32–1.41)
Spinal stenosis	0.66 (0.38–1.16)	0.32 (0.08–1.23)
Deformity	0.77 (0.30–1.99)	0.38 (0.11–1.28)
Postoperative conditions	1.35 (0.63–2.90)	0.53 (0.11–1.28)
Previously undergone spinal surgery (%)	0.58 (0.38–0.88) ^**a**^	1.05 (0.51–2.18)
Long fusion (> 2 levels)	0.78 (0.46–1.34)	1.05 (0.42–2.65)

a indicates statistical significance

## Discussion

In this prospective analysis of 348 consecutive patients undergoing elective lumbar spine fusions, we explored return to work rates and determined which factors affected return to work. Overall, the return-to-work rate after lumbar spine fusion was reasonable, as 69% had resumed working at the 12-month follow-up and 76% at the 24-month follow-up. The predominant symptom did not affect return to work, but the type of occupation had a notable impact, as patients in physically demanding work were less likely to return to work than patients in light work. While the predominant symptom did not singularly influence the return to work, its confluence with the physical demands of occupation did. The return to work at 2 years was lowest in patients with predominant leg pain in physically demanding work (60%) and in patients who were not working preoperatively (55%).

Return-to-work rates of our study were in line with previous reports. Lee et al. [3] reported a similar return to work of 75% within 1 year of lumbar spine fusion, but in a much smaller patient sample (n = 52) and a retrospective setting. Anderson et al. [1] studied patients undergoing anterior lumbar interbody fusion for chronic low back pain in a prospective setting, and reported return to work as high as 92%, when including only patients actively working at the time of surgery (n = 49). Results in small samples are easily biased, adding uncertainty to comparisons of results. 2 prospective studies on elective lumbar spine surgery reported return to work of 82% in a short, 3-month follow-up [14], and 75% in a 12-month follow-up [17]. However, only 26–36% of surgeries were fusions in those studies, while others were decompressions [14,17]. DiGiorgio et al. [18] reported a similar return to work of 77% in patients undergoing single-segment surgery for degenerative lumbar spondylolisthesis, but their surgeries were not fusion procedures.

Comparing return to work across countries is challenging because of variations in work practices, welfare, and social care. Studies from North America typically report rates of return to work at 75–90% in patients undergoing elective spinal surgery [1,12,14,17,28]. The differing results could stem from diverse welfare, differences in healthcare funding, the connection between health insurance and employment, and worker’s compensation programs. For example, Anderson et al. [29] report poor results of return to work after fusion for spondylolisthesis at 30% in patients with worker’s compensation claims. Similarly, Nguyen et al. [13] report poor return-to-work results at 2-year follow-up (26%) for worker’s compensation subjects undergoing lumbar fusion.

The common age of retirement in Finland is 65 years, so we excluded patients older than this to have a cohort available to the workforce. The study by Asher et al. [14] included patients of a similar age range with a similar return to work of 82%. Khan et al. [12] included only patients employed at the time of surgery and reported a slightly higher return to work of 85%. Most studies included patients older than ours, likely influencing results of return to work, reported at 75–89% [17,18,28].

Patients in physically demanding work were less likely to resume working than patients in light work. In previous studies the type of occupation, and specifically higher physical workload, have been shown to decrease the likelihood of return to work [12,14,15,17]. In contrast to our study, those studies have had a shorter follow-up of 3 months [14], were retrospective analyses [12], or had no requirement for all patients to be working before surgery [15]. A study by Devin et al. [17] reported that type of occupation was a top predictor for return to work, although in over half of the patients (55%) the type of occupation was unknown. It is possible that patients with heavier jobs had more severe back pathologies and therefore were less likely to resume working, although this cannot be explicitly verified by our analyses. Restoring working ability after lumbar spine fusion surgery is more difficult in patients in physically demanding work. With employer involvement, the alteration of job duties to physically less demanding alternatives should also be considered to promote return to work. Furthermore, the effect of other work-related factors on return to work has been studied previously. Schade et al. [30] report that occupational mental stress was a significant predictor of return to work in lumbar discectomy patients, whereas Den Boer et al. [31] reported job satisfaction to be only modestly associated with reduced work capacity after lumbar disc surgery, and concluded that physical work-related factors may be of more importance to work capacity. Our study did not include such psychosocial factors, but in future studies the importance of occupational mental stress or job satisfaction should also be considered, as it has been hypothesized that patients unsatisfied with their work could use the postoperative restrictions on daily activities as an excuse for not resuming work [30].

Conventionally it has been suggested that patients with predominant leg pain fare better after lumbar spine surgery [12,14,17-19,32], although some contradictory findings have also been published [33]. Our study showed that return to work rates were similar regardless of predominant back or leg pain. Dichotomizing patients based on surgeon-determined symptom predominance can be seen as arbitrary, as most patients had both back and leg complaints. Surgeon-determined classification, however, appeared valid as it aligned with patient-reported pain intensities (VAS back pain vs VAS leg pain). Results of surgery are highly dependent on patient selection. Many surgeons are hesitant to proceed to fusion with back pain dominant symptoms. Our findings support the fact that with careful patient selection and a proper indication for lumbar spine fusion, the predominant location of pain does not necessarily influence the outcome of surgery in terms of return to work, and therefore patients should not be excluded from surgery based on their symptom predominance. Differences in background pathologies may explain the different return to work between the leg pain and back pain groups. Patients in the leg pain group had more severe pathologies, with more symptoms of nerve compression and claudication. Indications in the back pain group were more benign, and their surgeries were more often their first fusion. In our study, history of previous spinal surgery had an adverse effect on return to work in the leg pain group.

In our study, patients not working preoperatively were less likely to resume working than patients who were preoperatively working. This finding is widely supported by previous studies, where disengagement in work preoperatively has been found as a strong risk factor for not resuming work after lumbar spine surgery [1,3,12,13].

Socioeconomic group and physical demands of work were the greatest factors associated with return to work in our study. The likelihood of return to work in the leg pain group was higher in upper-level employees and patients with lower baseline ODI. Several studies have made similar associations regarding ODI and return to work [12,14,18], but in a shorter follow-up time [14], including patients with a worker’s compensation claim [12,14] or in a smaller patient sample [18]. In the back pain group, return to work was similar regardless of the physical demands of occupation. Younger age and upper-level occupation were associated with increased return to work in line with previous reports [12,15,18].

### Strengths

The main strength of this study is based on prospectively collected large data of consecutive patients undergoing lumbar spine fusion. In addition, the study sample represents a population-based cohort of lumbar spine fusion patients with real-life data, in a national health insurance-based system.

### Limitations

The patient selection process in this study with no predefined indications for fusion can be seen as a limitation but also a strength of this study. It should be noted that this approach reflects the typical patient cohort undergoing lumbar spine fusion, without special exclusions. It is also noteworthy that our study lacks data on work capacity, making it plausible that all patients may not have returned to work at their full preoperative capacity. The return-to-work data was collected from medical records and could not be validated from national registries, as these do not exist in Finland, which we consider a minor limitation.

### Conclusions

Three-quarters of patients returned to work successfully after elective lumbar spine fusion. The preoperative dominance of leg or back pain alone was not associated with return to work. Patients in physically light work and in a higher socioeconomic group were more likely to return to work, whereas physically demanding work decreased the likelihood of return to work, especially in patients with predominant leg pain.

## Notes

All authors are responsible for conceptualizing the study. JL wrote the first draft of the manuscript. HK, JL, and LT performed the statistical analyses. All authors critically reviewed the drafts and accepted the final version of the manuscript. 

Handling co-editors: Paul Gerdhem and Philippe Wagner

*Acta* thanks Niyaz Hareni and Ivar Rossvoll for help with peer review of this manuscript.
